# Low-profile double plating versus dorsal LCP in stabilization of the olecranon fractures

**DOI:** 10.1007/s00402-020-03473-9

**Published:** 2020-05-16

**Authors:** Stefanie Hoelscher-Doht, A.-M. Kladny, M. M. Paul, L. Eden, M. Buesse, R. H. Meffert

**Affiliations:** 1 Department of Trauma, Hand, Plastic and Reconstructive Surgery, University Hospital of Wuerzburg, Oberduerrbacher Strasse 6, 97080 Wuerzburg, Germany; 2 Institut Straumann AG, Peter-Merian-Weg 12, 4052 Basel, Switzerland

**Keywords:** Olecranon, Plate, Biomechanical, Fracture, Low profile

## Abstract

**Introduction:**

Proximal ulna fractures are common in orthopaedic surgery. Comminuted fractures require a high primary stability by the osteosynthesis, to allow an early functional rehabilitation as fast as possible, to reduce long-term limitations of range of motion. Classical dorsal plating is related to wound healing problems due to the prominence of the implant. New low-profile double plates are available addressing the soft tissue problems by positioning the plates at the medial and lateral side. This study analysed whether, under high loading conditions, these new double plates provide an equivalent stability as compared to the rigid olecranon locking compression plate (LCP).

**Materials and methods:**

In Sawbones, Mayo Type IIB fractures were simulated and stabilized by plate osteosyntheses: In group one, two low-profile plates were placed. In group two, a single dorsal plate (LCP) was used. The bones was than cyclically loaded simulating flexion grades of 0°, 30°, 60° and 90° of the elbow joint with increasing tension forces (150 , 150 , 300  and 500 N). The displacement and fracture gap movement were recorded. In the end, in load-to-failure tests, load at failure and mode of failure were determined.

**Results:**

No significant differences were found for the displacement and fracture gap widening during cyclic loading. Under maximum loading, the double plates revealed a comparable load at failure like the single dorsal plate (LCP). The double plates failed with a proximal screw pull-out of the plate, whereas in the LCP group, in 10 out of 12 specimens the mode of failure was a diaphyseal shaft fracture at the distal plate peak.

**Conclusion:**

Biomechanically, the double plates are a good alternative to the dorsal LCP providing a high stability under high loading conditions and, at the same, time reducing the soft tissue irritation by a lateral plate position.

## Introduction

Proximal ulna fractures account for 10% of all fractures of the upper extremity. In 20% of the fractures of the olecranon, comminuted fractures, instability or the combination of both as in dislocated fractures occur [1, 2]. Those more complex proximal ulnar fractures are related to a direct trauma or due to metaphyseal bone loss as in osteoporosis [3]. A conservative treatment is rarely indicated and reserved for stable, non-dislocated fractures, which do not require a longer immobilization phase and can be treated with an early functional physiotherapeutic rehabilitation. Simple fractures of the proximal part of the olecranon are usually treated operatively with tension band wiring. Olecranon fractures of the distal part of apex of the articular surface need to be fixed with a more stable plate osteosynthesis. Also, more complex fractures with instability and comminuted fractures require a high stability by the fixation. For those fractures, a tension band wiring does not provide enough stability and in usual clinical practise locking plates are used [4]. They are placed dorsally at the ulna to resist the bending forces by the triceps tendon [4, 5]. An often-used plate type is the locking compression olecranon plate (LCP) from DePuy Synthes, Johnson & Johnson, USA, which is placed directly dorsally from the apex of the olecranon to the diaphysis.

General complications are a high risk of wound healing problems especially in older patients and in patients with an extensive soft tissue trauma, and the need of a revision with implant removing due to irritation even after the primary healing [4]. New low-profile plates are actually available, which are placed lateral at the olecranon. In clinical practise, there are advantages of these plates like exact anatomical shaping and reduction of soft tissue irritation. Also, the options of placement of the screws from two sides in 90° angle to each other in double-plate osteosynthesis are described as very helpful in clinical practise [6, 7]. To determine the biomechanical stability of these new low-profile locking plates for the proximal ulna, a biomechanical evaluation is needed. In a first biomechanical study with a low loading protocol, these new implants demonstrated very promising first results compared to a dorsal single plate [8]. Further investigations are needed analysing the biomechanical properties of the low-profile plates under higher loading conditions. Therefore, in this study, a double low-profile plate osteosynthesis was biomechanically compared to a high frequently clinically used dorsal single plate under loading conditions simulating daily life activities. A systematic analysis of the biomechanics of the elbow by Berme and Nicol et al., revealed forces around 300 N acting in the elbow joint during clothing or using cutlery. Getting out of a chair with the help of the upper extremities results in an effective force of 1700 N in the medial elbow compartment, and, especially the incisura trochlearis is affected with 20 times higher forces than a weight scales in the hand [9, 11].

We hypothesized, that even compared to a single dorsal plate (LCP, DePuy Synthes, Johnson & Johnson, USA) with a screw thread in plate thread system, which provides a very high stability, the new low-profile locking plates (Olecranon plates 2.8 mm, Medartis^®^, Switzerland) placed at the lateral side of the proximal ulna provide biomechanically an equivalent stability in comminuted olecranon fractures.

## Materials and methods

**Fig. 1:**
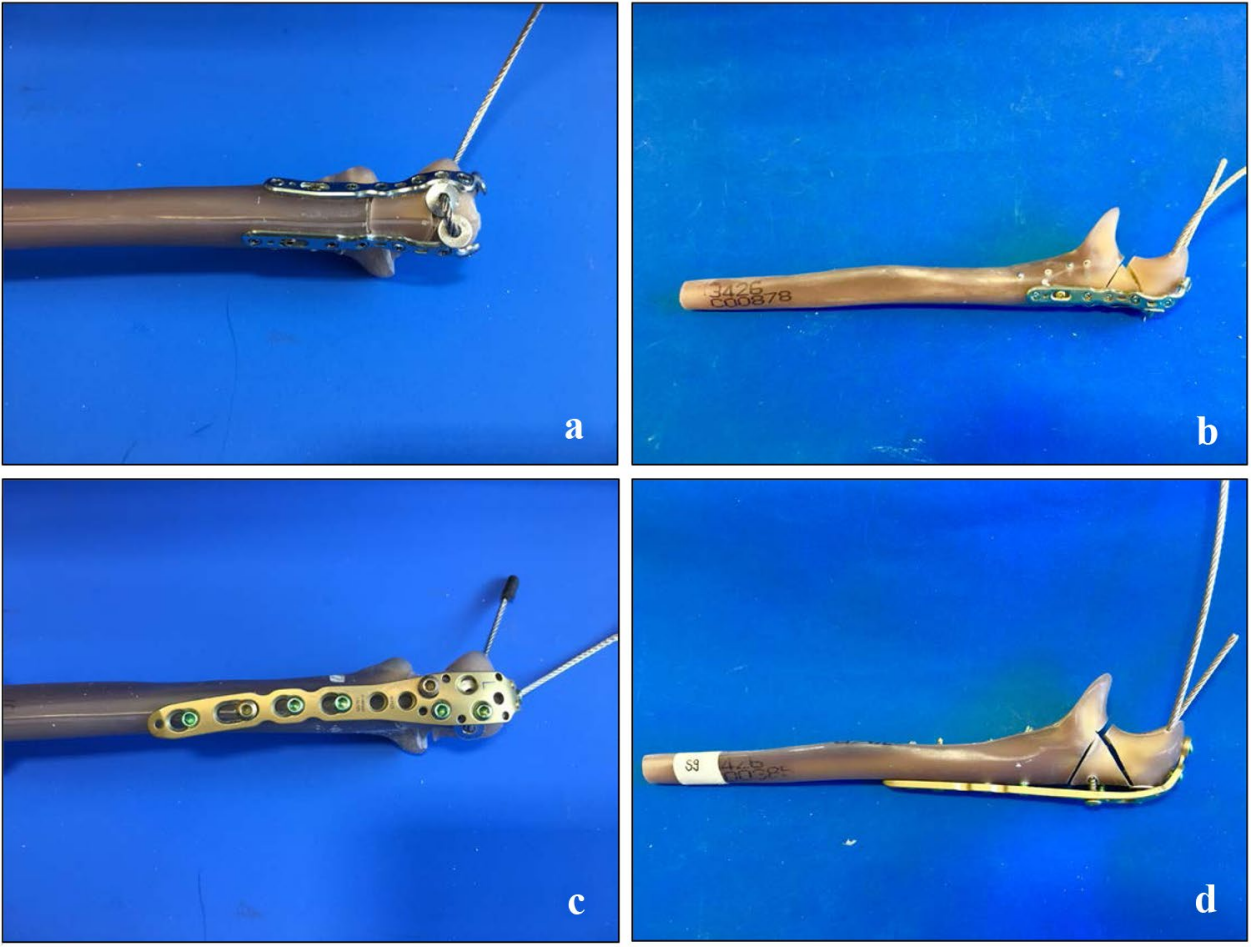
Fracture simulation and stabilization. In synthetic bones, comminuted olecranon fractures type Mayo IIb were simulated and stabilized by low-profile double plates (**a, b**) or in group two, by a single dorsal LCP (**c**, **d**)

Comminuted olecranon fractures (type Mayo IIb) [11–13] were created by an osteotomy in synthetic ulnar bones (Sawbones 3426, Sawbone^®^, Sweden) [14, 15]. Therefore, the bones were clamped in a custom-made device and the osteotomy was performed by an oscillating saw (PARKSIDE^®^, Germany) [12]. The fractures were then stabilized in two different ways: in group one, a double-plate osteosynthesis with low-profile locking plates (curved proximal ulnar plates 2.8, Medartis^®^, Switzerland) was used (Fig. 1a, b). In group two, an olecranon locking plate (LCP 3.5, DePuy Synthes, Johnson & Johnson, Germany) was placed dorsally at the olecranon (Fig. 1c, d. The low-profile locking plates were contoured to the bone without damaging a screw hole by the usual devices served by the company. The position of the plates was performed according to manufactural recommendations: the double plates were fixed at both sides of the proximal ulna with angle-stable screws placed in 90° angle to each other from both sides. Distally, the two plates ended alternated to each other. The proximal fracture fragment was fixed with four angle-stable screws, the wedge fracture fragment with three screws and the distal ulna with seven screws like in usual clinical practise and recommended in literature [7]. In group two, no contouring of the plate to the bone was needed. The LCP was placed ‘classically’ at the dorsal side of the ulna with three angle-stable screws in the proximal, two screws (one angle-stable) in the wedge and four in the distal fracture fragment. Screw length and placement in both groups followed the recommendation of both manufacturers with attention to regular fixation of all fracture fragments and no interaction between the distal shaft screws and the axially to the ulna shaft placed screws from the apex of the olecranon. All osteosyntheses were performed by the same senior orthopaedic surgeon and correct implant positioning was verified radiologically in all specimens.

A total of 24 ulnae were tested. Group size was calculated in a biometrical report by the Institute of Clinical Epidemiology and Biometry of the University of Wuerzburg, Germany.

For biomechanical testing, the main acting force of the triceps tendon needed to be simulated: Related to Koslowsky et al. [16], two parallel holes were drilled at the apex of the olecranon and a 2.0 mm special steel wire (Hamburger Tauwerke GmbH and Co. KG, Germany) providing a high flexibility and stability was inserted u-shaped. To hinder a cutting through the bone during testing, the wire was secured by flat washers distributing the force on the sawbone [14]. The flat washers did hinder the screw setting of one hole in the proximal fracture fragment in the LCP group. By the lateral position of the double plates, the positioning of the screws was not affected by the washers for this group.

**Fig. 2:**
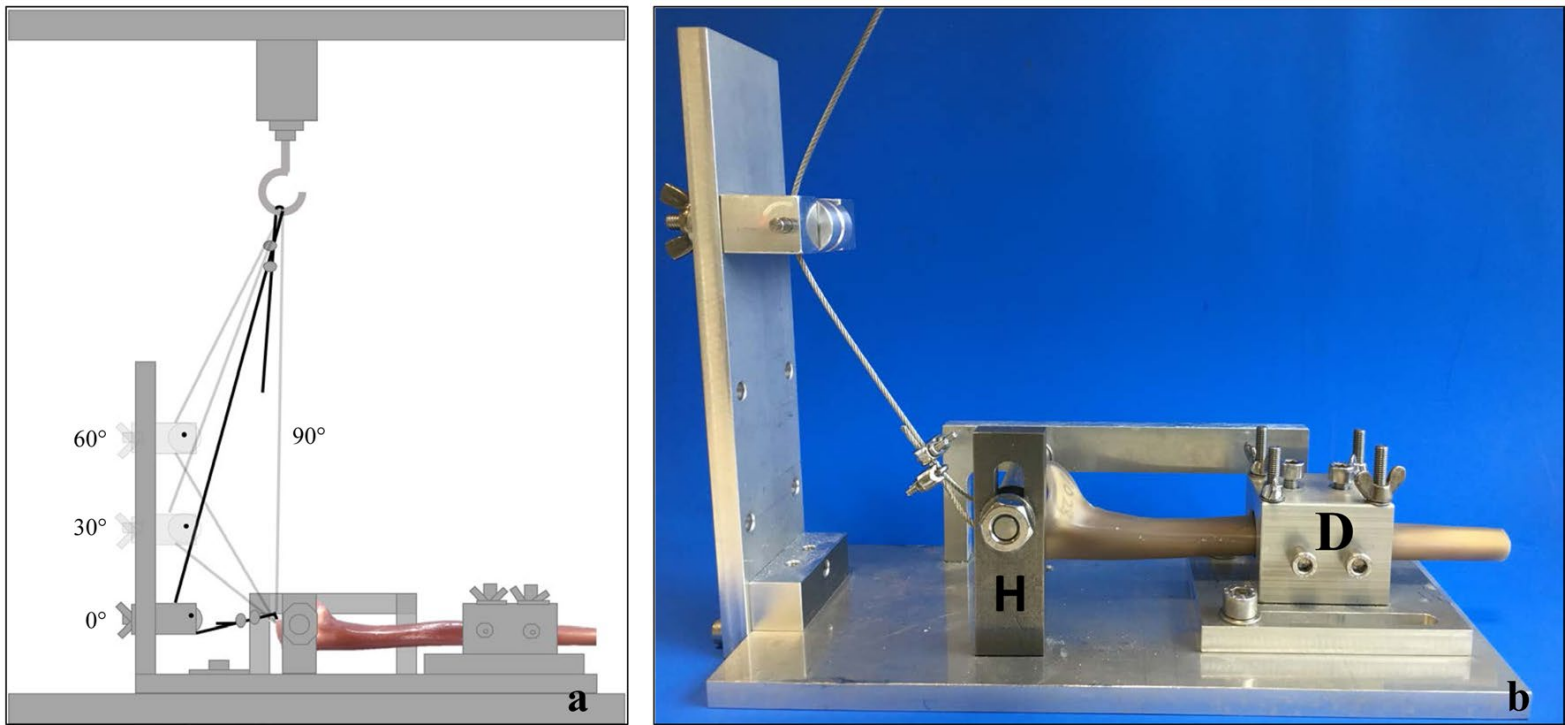
Biomechanical test set-up. Triceps tendon forces were simulated in 0°, 30°, 60° and 90° flexion (related to the shaft axis of the ulna) in the material testing machine Zwick Roell Z020 (**a**). The diaphysis of the ulna was fixed in a custom-made device (**b**) and an installed perpendicular to the longitudinal axis of the ulna formed a hypomochlion (**c**)

For the biomechanical testing, the bones were shortened to 210 mm from the apex of the olecranon. After fracture simulation and stabilization, the bones were clamped horizontally in a special device in the material testing machine Zwick Roell Z020 (D in Fig. 2). To reproduce the trochlea, a tensioner (20 mm diameter) was installed perpendicular to the longitudinal axis of the ulna as a hypomochlion [16, 17] (Fig. 2). In a pre-testing series, the test set-up was evaluated: According to former biomechanical studies, different flexion grades of the elbow joint were simulated [12, 18]. In 0°, 30°, 60° and 90° flexion, tension forces (150 N, 150 N, 300 N and 500 N) were applied cyclically like described in detail below and, in the end, in 90° flexion load-to-failure tests were performed (Fig. 2). The forces were orientated on the actual literature and on the forces acting on the proximal ulnae during joint motion in daily life [14, 16, 17, 19, 20]. At first, in 0° flexion 10 settling cycles were done from 5 N to15 N in the unloading and loading phase with a speed of 25 mm/min. Then, 500 measuring cycles from 10 to 150 N in 0°, followed by 300 measuring cycles from 10 to 150 N in 30°, 300 measuring cycles from 20 to 300 N in 60° and 400 measuring cycles from 20 to 500 N in 90° were performed. In the end, in 90° flexion, the bones were loaded till failure. The test set-up in detail is shown in Table 1.

**Table 1:** Sequence of the biomechanical test set-up

Test phase		Flexion angle in degree	Interval of tension force in *N*	Number of cycles
Cyclic	Settling cycles	0	5–15	10
Cyclic	Loading phase	0	10–150	500
		30	10–150	300
		60	20–300	300
		90	20–500	400
Static	Load-to-failure test	90	–	–

10 settling cycles from 5 to 15 N were followed by a cyclic loading phase in 0°, 30°, 60° and then 90° induction of the load. In every flexion grade, the number of applied cycles and the range of loading are described. After the cyclic loading phase, the specimens were loaded until failure in 90° flexion

**Fig. 3:**
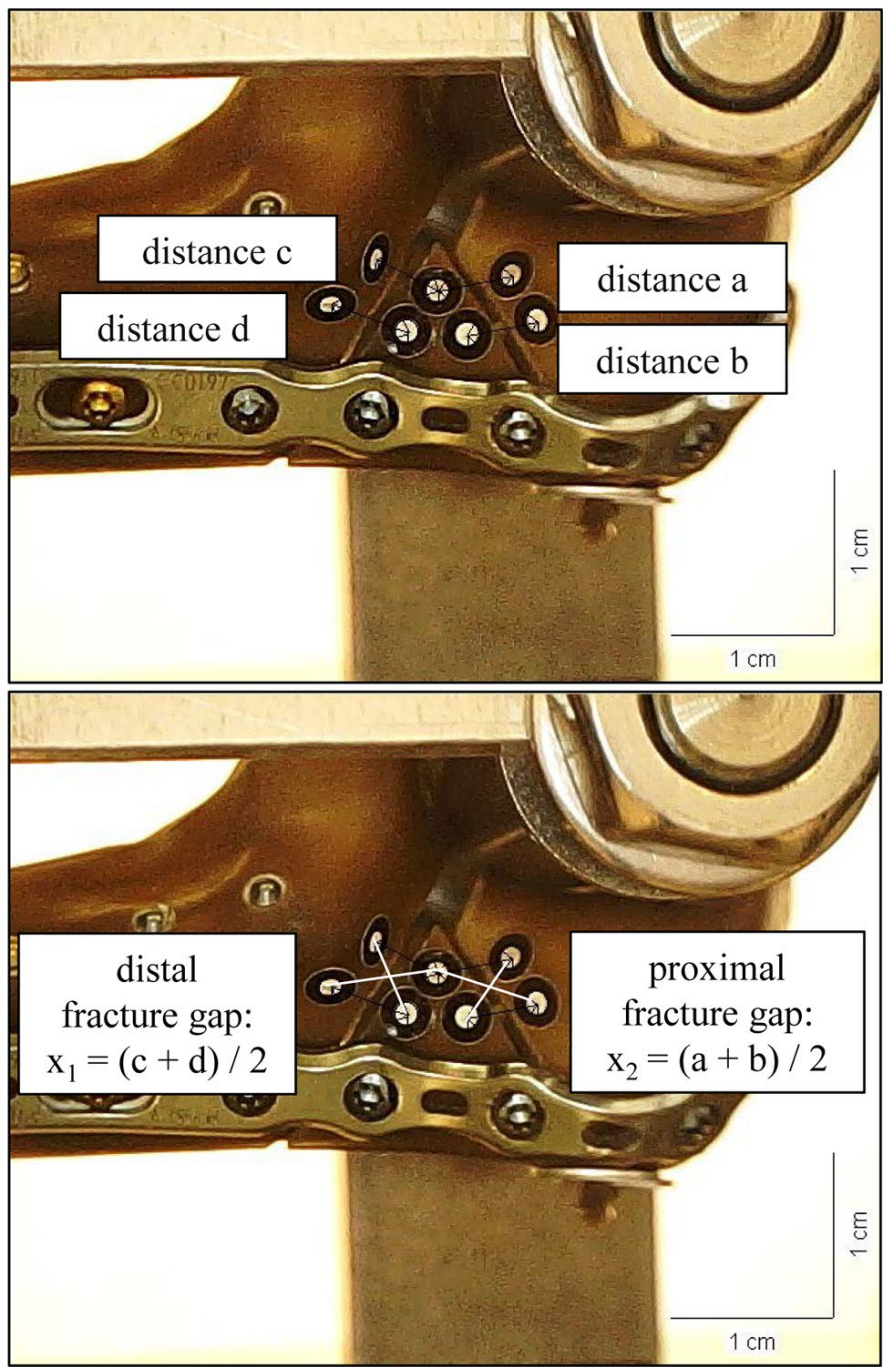
Recording of the relative fracture gap movement. Fracture gap movement was tracked by video capture synchronization of a camera microscope (DigiMicro Profi dnt^®^). One picture was taken prior to cyclic loading to determine the scale. In test cycle five and the last cycle during the loading phase of cyclic loading two more pictures were taken in every flexion grade. Like described before, fracture gap movement was calculated between the placed markers as mean value *X*_1_ (*X*_1_ = [a + b]/2) and value *X*_2_ (*X*_2_ = [c + d]/2) referring to Nowak et al. [15, 22, 23]

The displacement during cyclic testing was recorded by the traverse of the material testing machine and fracture gap movement was tracked by video capture synchronization of the camera microscope (DigiMicro Profi dnt^®^). One picture was taken prior to cyclic loading to determine the scale. In test cycle five and the last cycle during the loading phase of cyclic loading two more pictures were taken in every flexion grade. Like described before, fracture gap movement was calculated between the placed markers as mean values *X*_1_ (*X*_1_ = [a + b]/2) and *X*_2_ (*X*_2_ = [c + d]/2) (Fig. 3) [18]. In the load-to-failure tests, the load at failure and the mode of failure were determined.

The statistical analysis of the results was done in cooperation with the Institute of Clinical Epidemiology and Biometry of our university. With SPSS^®^ Statistics 23, the data were analysed for normal distribution (Shapiro–Wilk test) and for significant differences (*p* < 0.05) with the Levene test or Mann–Whitney *U* test.

## Results

### Cyclic loading phase

**Fig. 4:**
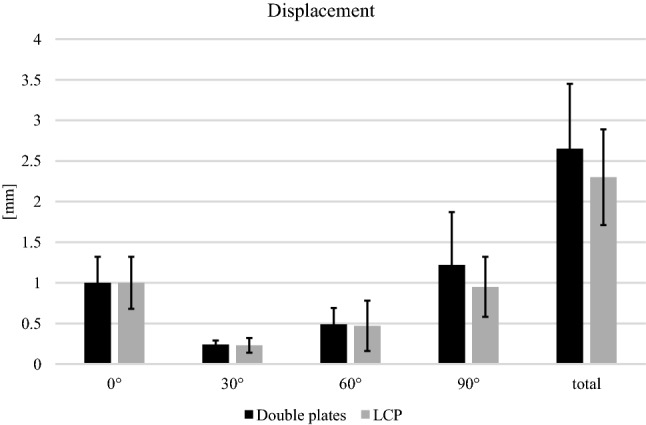
Displacement. No significant differences were determined between the groups in any flexion grade (0°: *p* = 0.997, 30°: *p* = 0.868, 60°: *p* = 0.551, 90°: *p* = 0.219, total displacement: *p* = 0.301)

**Fig. 5:**
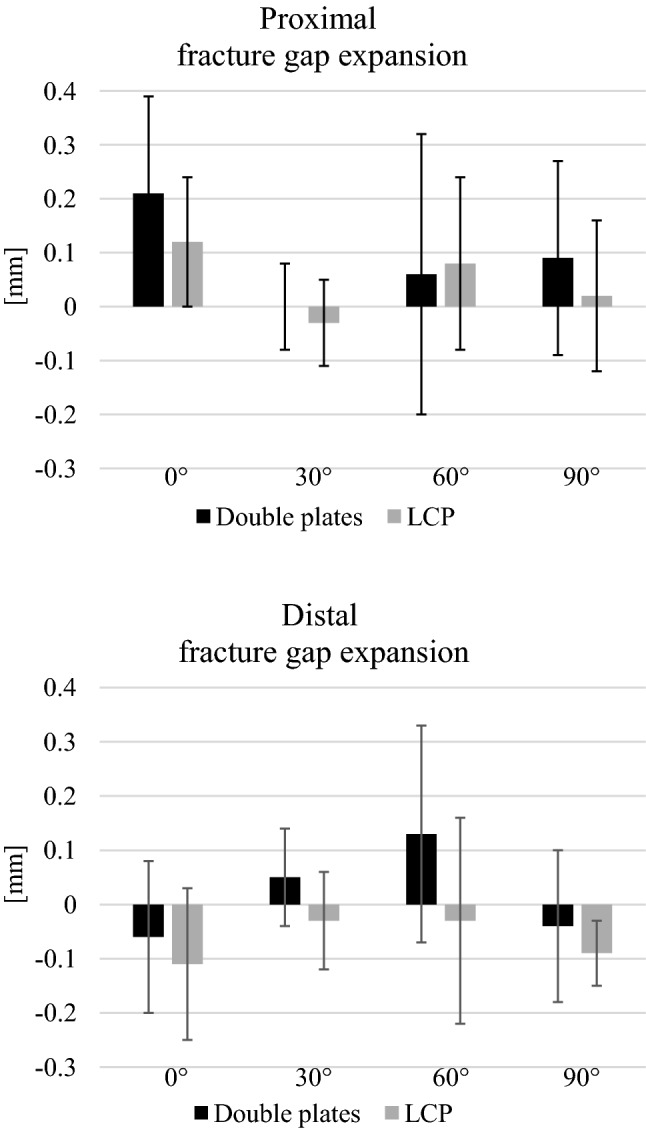
Relative fracture gap movement. The main fracture gap expansion was seen for 0° for the proximal and in 60° for the distal fracture gap. The proximal fracture gap expanded mostly, whereas the distal fracture gap was more compressed and so negative values were determined. No significant differences in-between groups are found for both fracture gaps (Proximal: 0°: *p* = 0.158, 30°: *p* = 0.418, 60°: *p* = 0.893, 90°: *p* = 0.114) (Distal: 0°: *p* = 0.336, 30°: *p* = 0.080, 60°: *p* = 0.075, 90°: *p* = 0.308)

No significant differences were found for the displacement for any flexion grade (0°, 30°, 60° and 90°) and for the total displacement during the cyclic loading phase (Fig. 4). The relative movement of the fracture gap proximal and distal for both osteosyntheses is shown in (Fig. 5). No significant differences between both groups were detected.

### Static loading phase

**Fig. 6:**
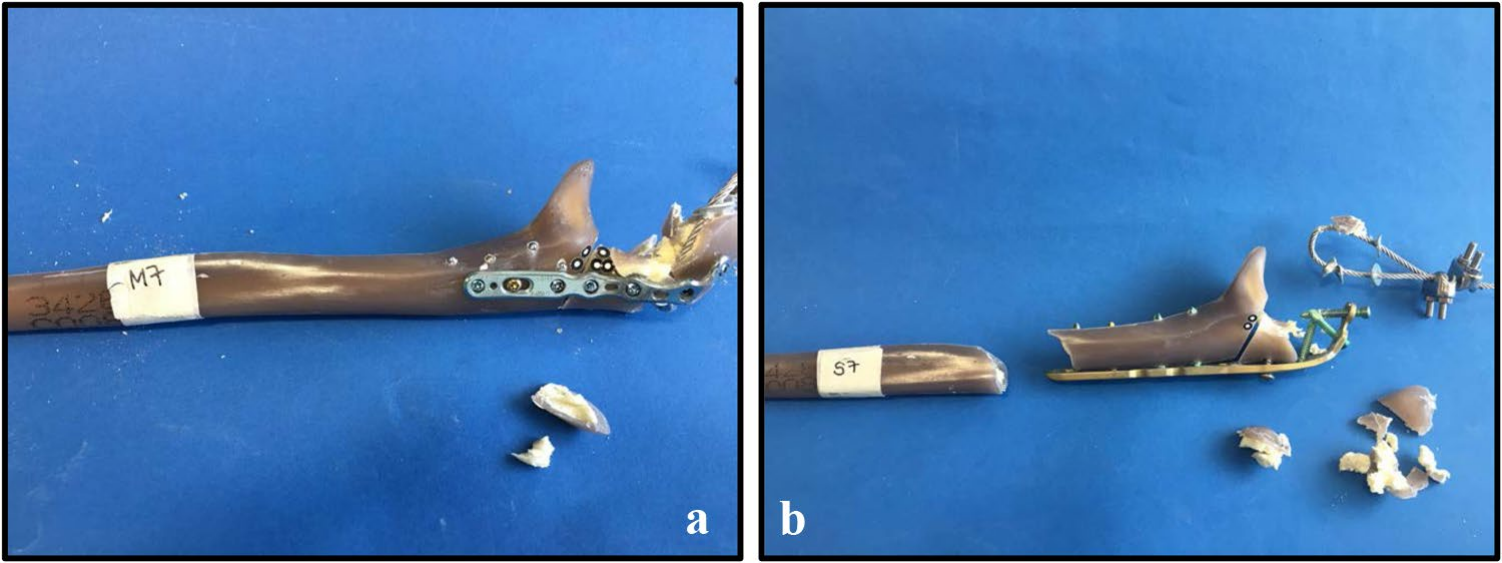
Mode of failure. The mode of failure under maximum loading of the specimens is shown. The double plates failed with a proximal screw pull-out of the plate (**a**), whereas in the LCP group, in ten out of twelve specimens the mode of failure was a diaphyseal shaft fracture at the distal plate peak (**b**). In the double-plate group, none failed by a shaft fracture

**Fig. 7:**
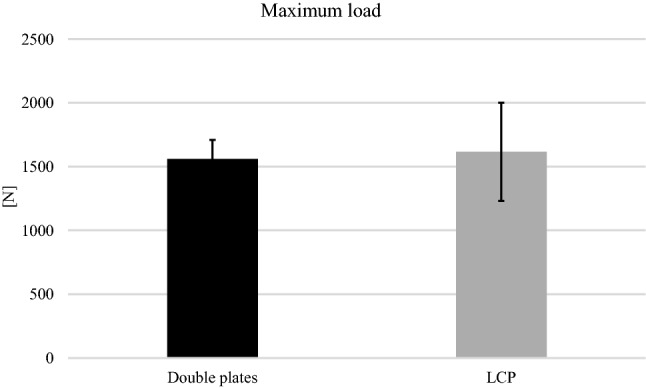
Maximum load. The maximum load of both groups in the load-to-failure tests. No significant difference was found (*p* = 0.651)

All specimens survived the cyclic loading phase and underwent the load-to-failure tests. In the LCP group, in 10 out of 12 specimens the mode of failure was a diaphyseal shaft fracture at the distal plate peak (Fig. 6b), whereas in the other group the proximal screws were pulled out of the plate (Fig. 6a). The load at failure revealed no significant differences (*p* = 0.65) for the low-profile double plates (1560.83 N 148.05 N) and the LCP (1615.83 N 384.32 N) (Fig. 7).

## Discussion

Multi-fragmentary olecranon fractures require a high stability by the osteosynthesis to hold the fracture fragments in a reduced position. An early postoperative functional rehabilitation is regularly performed to achieve the best postoperative result of range of motion. To realize a high primary stability, often plate osteosyntheses are used. To reduce postoperative wound healing problems, the new low-profile plates are an interesting alternative to the classic dorsal plates. Katthagen et al., described in an actual clinical review advantages like reduced soft tissue irritation and better retention of small joint fragments for the use of double plates in complex olecranon fractures [21]. Discussed disadvantages like a reduced blood supply by double plating of fractures of the upper extremity are decreased by modern low contact plate design and are not seen as a big problem at the olecranon in clinical practise. Anyway, a good blood perfusion of the ulna is given by the artery ulnaris [22]. Exposure of the bone is usually related to the complexity of the fracture and in multi-fragmentary fractures no additional soft tissue preparation is needed for a double plate osteosynthesis, whereas in simple olecranon fractures, a more invasive soft tissue preparation is not avoidable. Especially for the primary stability, the advantages like higher stability and anchoring sites of double plating appear to outweigh potential disadvantages [23, 24].

Lots of biomechanical studies analysed simple olecranon fractures and their fixation with tension band wiring, intramedullary locking nails, fibre wire fixation or cancellous screws [14, 17, 19, 20, 25, 26]. Studies, which simulated more complex proximal ulnar fractures, like in this presented study, are less frequent: intramedullary nail systems demonstrated a higher stability than the tension band wiring and the first-generation locking plates for the olecranon in comminuted fractures [11, 18, 27]. But the intramedullary nails not became widely accepted in daily clinical practise for fracture fixation in complex proximal ulnar fractures in the last years. In contrast, the locking compression plate (LCP, DePuy Synthes, Johnson & Johnson, USA) is clinically often used for olecranon fracture stabilization. In an actual biomechanical analysis, the LCP demonstrated a higher stability compared to the LCP hook plate [28]. The angle-stability is realized by a very rigid screw in screw system. A direct biomechanical comparison of this high frequently used implant to the new low-profile double plates is therefore interesting. The new low-profile plates were at the first time analysed in a clinical related biomechanical test set-up by Hackl et al. [8]. The double plates achieved in a low loading test protocol with a peak load of 80 N a comparable stability like the tested dorsal plate from Variax™, Stryker, Duisburg, Germany. In contrast to this study, we wanted to simulate higher loading conditions and to analyse the new low-profile plates compared to the LCP with a screw thread in plate hole thread locking system. The test set-up was chosen according to former biomechanical studies, to analyse the stability of the plates under different flexion and loading conditions [17, 19, 20, 29, 30]. Well in agreement with other studies, the both plate osteosyntheses used in this study showed the highest rigidity in 30° flexion, and the highest displacement and fracture gap movement in 0° and 90° flexion [8, 29]. Interestingly, in-between groups, we could not detect a difference under cyclic loading in any flexion grade and none of the specimens failed under cyclic loading. Also, for the load at failure, no differences were found.

All in all, the results demonstrated for higher loading conditions either cyclically or in load-to-failure tests a biomechanically equivalent stability for the low-profile plates compared to the very rigid LCP. Based on that, a clinical application of the double plates is a favourable alternative to classic dorsal plating. Of course, only the primary stability is considered in this study and fracture healing or soft tissue effects are not taken into account. Another limitation of this study is the use of synthetic bones, resembling bone of healthy adults. But the use of synthetic bones is an established method in biomechanical research, also in olecranon fracture analysis. Further investigations in bones with minor bone quality like osteoporotic bones are needed to determine the stability of the different plates also in this different situation. Moreover, a limitation of the study may be the use of washers. These hinder screw placement in the LCP, which could perhaps otherwise have provided a higher stability under cyclic loading. However, in our clinical experience, the fixation of the proximal fracture fragment with three 3.5 mm locking screws and fixation of the wedge fragment with a corticalis and one angle-stable screw provides sufficient stability. Different screw settings in both plates might affect the biomechanical results, and, could be explored in prospective studies.

## Conclusion

Biomechanically, the low-profile plates provide under high loading conditions a comparable stability like the very rigid, high profile LCP, and, considering advantages like reduced wound healing problems, are an interesting alternative to classic dorsal plating.
